# Lipid nanoparticle-delivered intrabodies for inhibiting necroptosis and pyroptosis

**DOI:** 10.1042/BCJ20253191

**Published:** 2025-08-20

**Authors:** Veerasikku Gopal Deepagan, Xiuquan Ma, Farzaneh Bazregari, Jiyi Pang, Jan Schaefer, Joanne M. Hildebrand, Ruby K. Dempsey, Marcel Doerflinger, Christopher A. Baldwin, Florian I. Schmidt, James M. Murphy, Ranja Salvamoser, James E. Vince

**Affiliations:** 1 The Walter and Eliza Hall Institute of Medical Research, Parkville, VIC 3052, Australia; 2 The Department of Medical Biology, University of Melbourne, Parkville, VIC 3010, Australia; 3 Mermaid Bio GmbH, München 81377, Germany; 4 Institute of Innate Immunity, Medical Faculty, University of Bonn, Bonn, Germany; 5 Core Facility Nanobodies, Medical Faculty, University of Bonn, Bonn, Germany; 6 Drug Discovery Biology, Monash Institute of Pharmaceutical Sciences, Parkville, VIC 3052, Australia

**Keywords:** inflammasome, lipid nanoparticle, MLKL, nanobody, necroptosis, pyroptosis

## Abstract

Intrabodies are intracellularly expressed high-affinity protein binders such as nanobodies and monobodies that offer an alternative approach to small molecules. However, the maturation of intrabody technology into new therapeutic modalities has been limited by the availability of a clinically relevant delivery system enabling sufficiently high levels of protein to be expressed in the cytosol. Here, we use lipid nanoparticle (LNP) systems based on clinically approved formulations for the efficient intracellular delivery of mRNAs encoding for intrabodies targeting mixed lineage kinase domain-like pseudokinase (MLKL) and apoptosis-associated speck-like protein containing a CARD (ASC), key mediators of the necrotic cell death modalities, necroptosis and pyroptosis, respectively. LNP delivery of intrabody mRNA resulted in robust protein expression, with an MLKL-binding intrabody preventing MLKL membrane translocation and protecting against necroptotic cell death. Similarly, LNP delivery of a bivalent intrabody targeting the inflammasome adaptor protein ASC protected against NLRP3 and AIM2 inflammasome-driven responses, including caspase-1 and IL-1β activation and gasdermin D-driven pyroptotic killing. These findings establish that LNPs harbouring anti-necrotic intrabody mRNAs allow for sufficient intracellular expression to neutralize necrotic cell death signalling and provide a general, clinically relevant, strategy for delivering therapeutic intrabodies into cells.

## Introduction

Antibody therapeutics are widely used to treat a broad spectrum of diseases, including cancer, autoimmune disorders and infectious diseases [1,2]. However, their application can be limited by several factors, including side effects such as acute anaphylactic reactions and cytokine release syndrome, poor tissue penetration due to their large size (~150 kDa) and high production costs [3]. Consequently, alternative antigen-binding scaffolds such as nanobodies (single-domain antibody fragments derived from camelids) and monobodies (based on the type III fibronectin domain) have been explored, with nanobody-based therapies targeting extracellular proteins, such as TNF, PD-L1 and von Willebrand factor approved for clinical use, and many others undergoing evaluation [4,5].

When compared with conventional antibodies, attractive unique features of nanobodies, monobodies and other protein binders (e.g. designed ankyrin repeat proteins) are their small size of ~15 kDa enabling access to ‘hidden’ epitopes, simple single-domain structure (i.e. can be encoded by a single DNA or mRNA), lack of a Fc region (thereby avoiding the potential for triggering anaphylaxis) and their stability in reducing environments [4,5]. These features also mean that single domain antibodies and antibody mimetics can fold properly inside cells to bind, with high affinities, their targets, resulting in the term ‘intrabodies’ to collectively describe their ability to function intracellularly [6]. Intrabodies have been extensively used as research tools, such as in live-cell imaging and as biosensors to help uncover new biology, although their capacity to inhibit proteins classified as undruggable highlights their potential as a new class of therapeutics [7]. For example, studies have demonstrated how intrabodies can block the signalling capacities of hard-to-drug targets, such as the cancer drivers RAS [8], β-catenin [9] and LMO2 [10], or key molecules required for programmed necrotic cell death pathways, necroptosis [11] and pyroptosis [12,13], implicated in numerous inflammatory conditions [14]. However, although a number of intrabody transporting systems, such as micro-injection, lentiviral transduction and the incorporation of cell-penetrating motifs, have been investigated [15], clinically proven modalities for the delivery of intrabodies into cells remain a major challenge for taking them forward as viable drugs.

Lipid nanoparticles (LNPs) represent a promising strategy for intracellular delivery of nucleic acids, including mRNA. These nanometre-sized, self-assembled vesicles protect mRNA from temperature variation, extracellular RNase degradation and premature clearance by the mononuclear phagocyte system or renal filtration [16,17]. LNPs safely transport mRNA across physiological barriers, predominantly via endocytosis, and deliver the payload into the cytosol [17]. Moreover, LNPs can be engineered for tissue-specific delivery by altering physicochemical properties such as lipid composition [18], surface charge [19], shape [16], particle size [20] and tagging with targeting ligands [21]. These modifications can enable LNP tissue and cellular trophism, thereby minimizing off-target effects. In addition, improved *in vitro* transcription (IVT) methods with optimized untranslated regions (UTRs), modified bases and effective 5′-cap structures have contributed to making LNP-mRNA technology a powerful tool for intracellular protein delivery that is bringing new biological medicines into the clinic [16].

Necroptosis and pyroptosis are two genetically encoded necrotic cell death pathways that evolved to counter pathogen infections, but their dysregulation coupled with their inflammatory nature has implicated them in driving numerous infectious, inflammatory and autoimmune diseases [14,22–24]. Mixed lineage kinase domain-like pseudokinase (MLKL) is the terminal effector of necroptosis, and when activated following death receptor, toll-like receptor (TLR) or Z-DNA-binding protein 1 signalling, it oligomerizes and unleashes the 4-helical bundle (4HB) domain, which then interacts with negatively charged membrane lipids to cause plasma membrane lysis and the release of immunogenic intracellular contents [14,25–27]. On the other hand, pyroptosis is initiated canonically through cytosolic pattern recognition receptors such as NLRP3, a sensor of cellular stress and potassium ion efflux; AIM2, a sensor of DNA; and Pyrin, an indirect sensor of bacterial toxins. When triggered, these inflammasome sensor proteins recruit the adaptor apoptosis-associated speck-like protein containing a CARD (ASC), which binds caspase-1. Subsequently, proximity-induced activation of caspase-1, for which ASC is essential [28], results in caspase-1 maturation of the inflammatory cytokines IL-1β and IL-18, and also processing of gasdermin D (GSDMD) [29,30]. The N-terminal domain of GSDMD subsequently forms plasma membrane pores, resulting in pyroptotic cell death and enabling the efficient release of activated IL-1β and IL-18 [31].

Despite the promise of targeting necroptosis or pyroptosis to treat diverse inflammatory-associated diseases, to date, no intracellular anti-necrotic drug has progressed beyond clinical trials. Here, we sought to leverage clinically approved LNP-mRNA technology to deliver intrabodies targeting two key mediators of necrotic cell death – an intrabody that targets MLKL, the terminal effector of necroptosis, and an intrabody targeting ASC, the common adaptor protein essential for pyroptosis downstream of inflammasome sensor protein activation. We demonstrate that LNP delivery of MLKL and ASC intrabody mRNA results in stable protein expression and potently blocks necroptosis and pyroptosis, respectively, paving the way for future trials examining their *in vivo* efficacy in relevant disease models.

## Results

### Inducible expression of MLKL and ASC intrabodies blocks necrotic cell death

**Figure 1:**
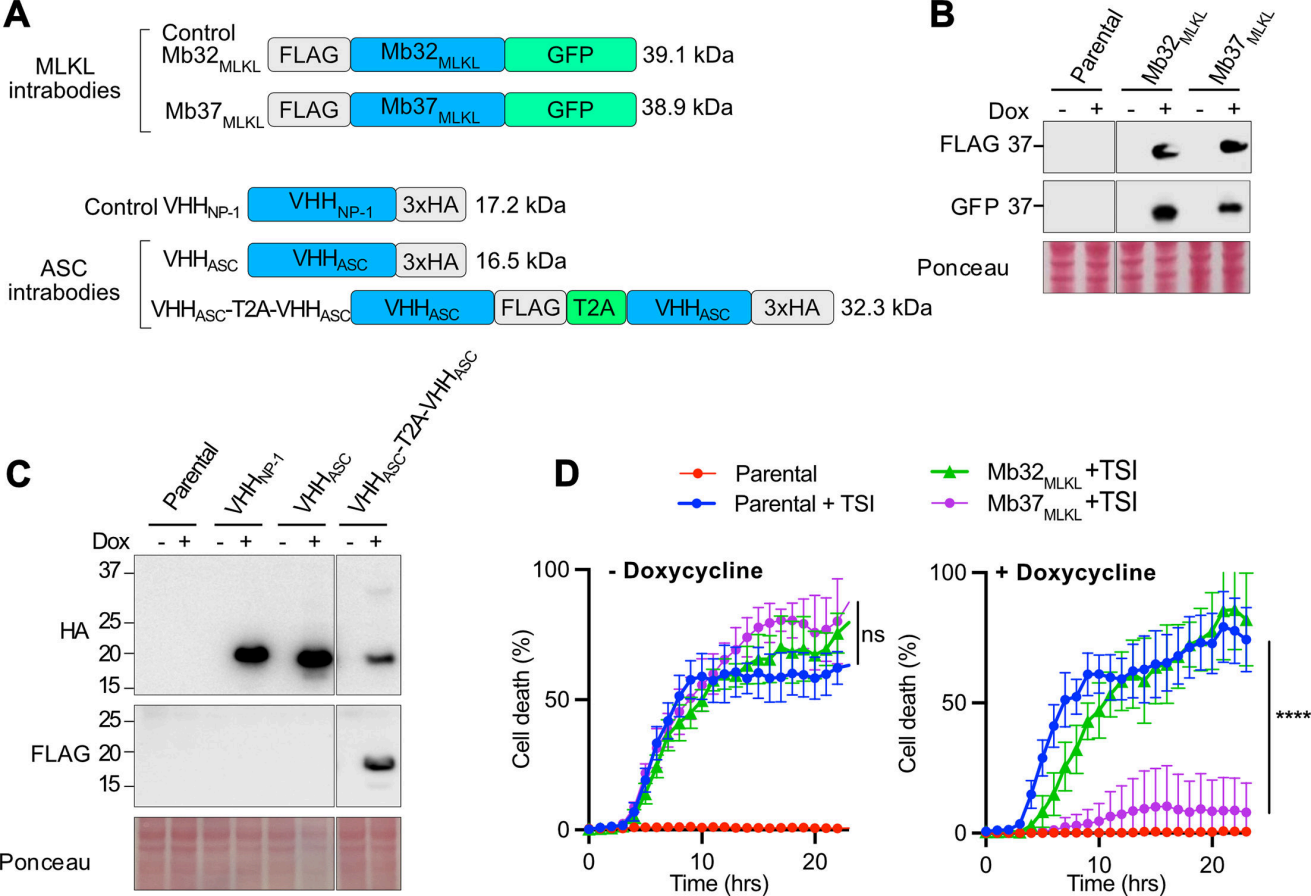
Doxycycline-inducible MLKL intrabody expression blocks necroptosis. (**A**) Schematic of doxycyline-inducible constructs. (**B**) Immunoblot analysis following overnight dox (100 ng/ml) treatment to induce expression of the indicated MLKL intrabodies in HT29 cells. (**C**) Immunoblot analysis following overnight dox (100 ng/ml) treatment to induce expression of ASC and NP-1 control intrabodies in iBMDMs. (**D**) Cell death kinetics of wildtype parental and stable intrabody containing HT29 cells following TSI (TNF (50  ng/ml), Smac mimetic Compound A (1  µM) and pan-caspase inhibitor IDN-6556 (10  µM)) stimulation with or without dox (0.1 µg/ml) pre-treatment overnight. Error bars represent the mean ± standard deviation (SD) from three technical repeats, *P*≤0.0001 (****), one-way ANOVA followed by Tukey’s multiple comparisons test. Data presented in B, C and D are representative of two independent experiments. ASC, apoptosis-associated speck-like protein containing a CARD; MLKL, mixed lineage kinase domain-like pseudokinase.

We first validated the anti-necrotic capacity of two published intrabodies, an MLKL inhibitory monobody, Mb37_MLKL_ [11], and ASC-targeting nanobody (VHH_mASC_) [32]. Mb37_MLKL_ binds the human MLKL 4-helical bundle domain (*Kd* 170 nM) that inserts into lipid membranes and is essential for necroptotic driven membrane lysis and cell death. As such, Mb37_MLKL_ blocks MLKL membrane translocation and cell killing but not the preceding steps in MLKL activation – its phosphorylation and oligomerization [11]. On the other hand, VHH_mASC_ selectively binds the pyrin domain of mouse ASC, required for ASC recruitment to inflammasome sensor proteins and inflammasome complex assembly, and thereby shuts down inflammasome responses [32]. We used Mb37_MLKL_ and a control monobody Mb32_MLKL_ that binds to, but does not inhibit, MLKL, FLAG-tagged at the N-terminus and fused with GFP at the C-terminus [11] (Figure 1A). The inhibitory VHH_mASC_ [32] was also tested as a self-separating fusion protein (VHH_mASC_-T2A-VHH_mASC_), with two tandem nanobodies modified with distinct HA and FLAG epitopes and split by a T2A sequence to enable their separation and detection. VHH_NP-1_ [33], a nanobody targeting the nucleocapsid of influenza A virus, was used as a negative control (Figure 1A). To assess intrabody expression, doxycycline (dox)-inducible stable HT29 cell lines containing MLKL intrabodies and immortalized bone marrow-derived macrophages (iBMDMs) cells harbouring ASC intrabodies were generated. Following induction with dox, immunoblot analysis confirmed successful protein expression of all intrabody constructs at the expected molecular weight (Figure 1B and C). Of note, the T2A motif containing tandem ASC intrabody showed excellent separation into two monomeric nanobodies, with only a faint band corresponding to the full-length ASC intrabody fusion detected (Figure 1C).

Next, the functional efficacy of MLKL targeting Mb37_MLKL_ was examined following overnight dox induction and subsequent treatment with the necroptotic stimulus, TSI: a combination of TNF, the Smac mimetic Compound A (Cp. A), and the pan-caspase inhibitor IDN-6556. Cell death analysis over time using an IncuCyte live cell imaging system demonstrated that, as expected, Mb37_MLKL_ expression abolished necroptotic cell death for more than 20 hours post TSI treatment, while the expression of the control intrabody, Mb32_MLKL_, did not (Figure 1D).

**Figure 2:**
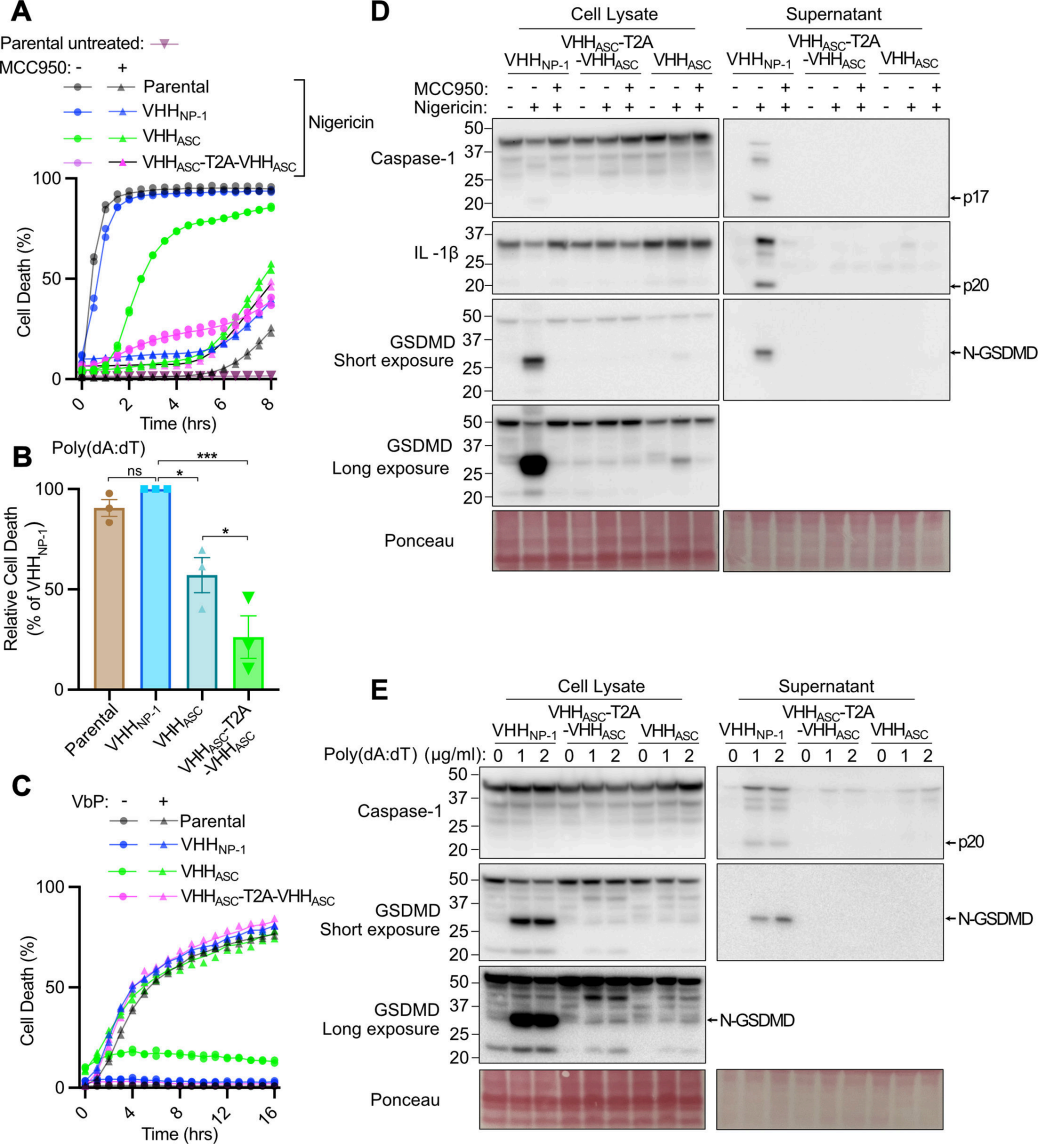
Doxycycline-inducible ASC intrabody expression prevents NLRP3- and AIM2-induced pyroptosis. (**A, B and C**) Wildtype parental and the indicated stable intrabody containing iBMDMs were pre-treated overnight with doxycycline (0.1 µg/ml) and cell death kinetics were monitored using IncuCyte imaging: (**A**) following stimulation with nigericin (5 µM) with or without MCC950 (10 µM) co-treatment; (**B**) after poly(dA:dT) (1 µg/ml) transfection; and (**C**) after VbP (2.5 µM) treatment. (**D and E**) Immunoblot analysis of pyroptotic signalling in total cell lysates and supernatants after (**D**) nigericin (5 µM) ± MCC950 (10 µM) and (**E**) poly(dA:dT) treatment (1 and 2 µg/mL). In (**B**), data are presented as mean ± SEM of pooled data from three independent experiments, *P*≤0.05 (*), *P*≤0.001 (***), one-way ANOVA Holm-Šídák’s multiple comparisons test. The IncuCyte data (**A and C**) symbols represent technical duplicates, and these experiements and the immunoblots (**D and E**) are representative results of two independent experiments. ASC, apoptosis-associated speck-like protein containing a CARD.

Pyroptosis and its ability to be inhibited by the ASC targeting intrabody was similarly evaluated by pre-treating iBMDM stable cell lines with dox overnight followed by nigericin stimulation to activate the NLRP3 inflammasome. Expression of monovalent VHH_mASC_ or the tandem VHH_mASC_-T2A-VHH_mASC_ intrabody protected cells from NLRP3-driven pyroptosis, while control VHH_NP-1_ intrabody-expressing cells, or wildtype parental iBMDMs, were not protected from nigericin killing (Figure 2A and Supplementary Figure S1). Notably, the VHH_mASC_-T2A-VHH_mASC_ intrabody exhibited stronger protection from pyroptosis than its monovalent counterpart and was comparable with the protection resulting from MCC950 treatment (Figure 2A), a selective and potent NLRP3 inhibitor [34]. Moreover, while MCC950 treatment conferred additional protection from nigericin-induced pyroptosis when used together with expression of the monovalent VHH_mASC_ intrabody (Figure 2A), it did not increase protection substantially when used in conjunction with the VHH_mASC_-T2A-VHH_mASC_ intrabody (Figure 2A). This indicates that the VHH_mASC_-T2A-VHH_mASC_ intrabody likely confers near-maximal protection from NLRP3 signalling and that the nigericin-induced death observed after ~5 hours of treatment in the presence of VHH_mASC_-T2A-VHH_mASC_ intrabody or MCC950 reflects NLRP3-independent killing. Therefore, while monovalent Mb37 intrabody suffices to shut down necroptosis signalling, a VHH_mASC_-T2A-VHH_mASC_ intrabody is required to efficiently inhibit pyroptotic cell death.

To further confirm the efficiency and specificity of monovalent and VHH_mASC_-T2A-VHH_mASC_ intrabodies, iBMDMs were treated with poly(dA:dT) to activate the AIM2 inflammasome that also requires ASC for pyroptotic killing or were stimulated with Val-boroPro (VbP), which triggers NLRP1-mediated pyroptosis that occurs independent of ASC in mouse cells [35]. Consistent with ASC intrabody blockade of NLRP3 killing, VHH_mASC_-T2A-VHH_mASC_ intrabody expression conferred optimal protection from AIM2-mediated pyroptosis when compared with the monovalent ASC intrabody (Figure 2B) but, as expected, neither ASC intrabody protected against NLRP1-mediated pyroptosis (Figure 2C).

Consistent with the analysis of NLRP3- and AIM2-driven cell death, immunoblots showed that NLRP3- and AIM2-induced processing (i.e. activation) of caspase-1, IL-1β and GSDMD was effectively shut down by ASC intrabody expression. The VHH_mASC_-T2A-VHH_mASC_ intrabody also better protected from GSDMD cleavage into its pore-forming N-terminal fragment (N-GSDMD) compared with VHH_mASC_ and acted similar to MCC950 treatment in the context of nigericin-induced NLRP3 triggering (Figure 2D and E). Together, these results demonstrate that the inducible expression of MLKL and ASC targeting intrabodies can effectively shut down necrotic cell death signalling.

### Efficient MLKL intrabody delivery, expression and inhibition of necroptosis using LNP-mRNA technology

**Figure 3:**
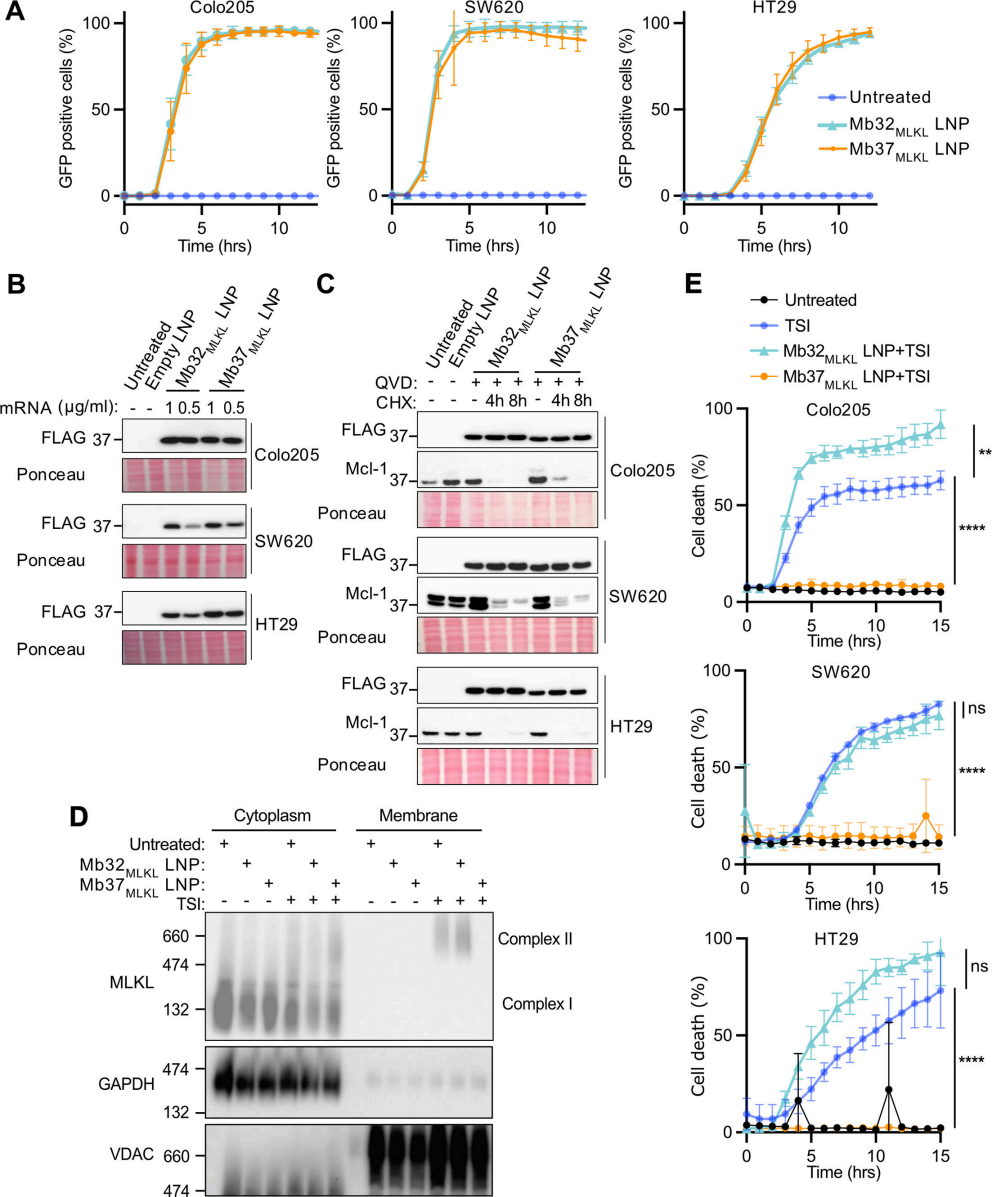
LNP-delivered MLKL intrabody mRNA prevents necroptosis by blocking MLKL membrane translocation. (**A**) Percentage of GFP-positive cells over time following LNP delivery of FLAG-MLKL-GFP intrabody mRNA (0.5 µg/ml) in Colo205, SW620 and HT-29 cells, measured using IncuCyte live cell imaging system. Error bars represent the SD of three technical replicates. See Supplementary Figure S3 for representative images. (**B**) Immunoblot of total cell lysates 24 hours post-LNP transfection using the indicated mRNA concentrations. (**C**) Immunoblot of total lysates from cells treated with LNPs (1 µg/ml mRNA equivalent) for 24 hours, followed by cycloheximide (CHX, 40 µg/ml) and Q-VD-OPh (QVD, 20 µM) treatment as indicated. (**D**) Analysis of MLKL oligomerization and membrane translocation via Blue Native PAGE after LNP delivery of the indicated MLKL intrabody mRNA (0.5 µg/ml mRNA equivalent) overnight followed by TSI (TNF (50  ng/ml), Smac mimetic Compound A (1  µM), pan-caspase inhibitor IDN-6556 (10  µM)) treatment for 6 hours. GAPDH and VDAC were used as cytoplasmic and membrane markers, respectively. (**E**) Cell death kinetics measured by IncuCyte imaging following TSI (TNF (50  ng/ml), Smac mimetic Compound A (1  µM), pan-caspase inhibitor IDN-6556 (10  µM)) treatment in cells that were pre-treated overnight with LNPs (0.5 µg/ml mRNA equivalent). Error bars represent the SD from three technical replicates, *P*≤0.01 (**),*P*≤0.0001 (****), two-way ANOVA followed by Tukey’s multiple comparisons test. All IncuCyte data (**A, E**) and immunoblots (**B, C, D**) are representative results of two independent experiments other than Figure 2E Colo205 cells where, in the independent experiment, no difference was observed between TSI-treated control and TSI-treated Mb32_MLKL_-expressing cells. LNP, lipid nanoparticle; MLKL, mixed lineage kinase domain-like pseudokinase.

Following our validation and optimization of anti-necrotic intrabodies using a stable cell line dox-inducible system (Figures 1 and 2), Mb32_MLKL_ control and Mb37_MLKL_ were cloned into an IVT vector incorporating an optimized synthetic 5′ UTR, a 2 x human β-globin 3′ UTR, and a poly(A) tail ( ≥ 128 A bases) [36] and mRNA synthesized. LNPs containing mRNA were formulated using SM-102 (ionizable lipid), 1,2-distearoyl-sn-glycero-3-phosphocholine (DSPC), cholesterol and 1,2-dimyristoyl-sn-glycero-3-methoxypolyethylene glycol (DMG-PEG) at a 50:10:38.5:1.5 molar ratio and encapsulated using a NanoAssemblr Ignite microfluidic system at an N/P ratio of 6. The physiochemical characterization of LNPs and mRNA loading was performed by dynamic light scattering (DLS) and Qubit analysis. This showed that LNPs were ~100 nm in diameter with a unimodal distribution and polydispersity index (PDI) <0.2, and no less than 85% mRNA encapsulation efficiency (Supplementary Figure S2 and Supplementary Table S1). Next, LNPs containing FLAG-Mb32_MLKL_-GFP and FLAG-Mb37_MLKL_-GFP mRNA were examined for their transfection efficiency in three necroptosis-susceptible cell lines (HT29, Colo205, and SW620) using GFP as a readout and measured by widefield fluorescence microscopy and IncuCyte live cell imaging. Robust GFP expression was observed across all cell lines, reaching ~100% within 12 hours of LNP-mRNA incubation (Figure 3A and Supplementary Figure S3A & S3B). Consistent with these data, immunoblotting showed an LNP-mRNA dose-dependent expression of FLAG-Mb32_MLKL_-GFP and FLAG-Mb37_MLKL_-GFP fusion proteins at their predicted molecular weights (Figure 3B). The intracellular stability of LNP-delivered MLKL intrabody protein was assessed by treating cells with the protein synthesis inhibitor cycloheximide. While the short-lived protein Mcl-1 rapidly degraded within 4 hours of cycloheximide incubation, MLKL intrabody protein remained stable for at least 8 hours across all cell lines (Figure 3C).

Next, the function of LNP-delivered MLKL intrabody was evaluated. Blue Native-PAGE analysis demonstrated that LNP delivery of Mb37_MLKL_, but not Mb32_MLKL_, blocked MLKL membrane translocation (Figure 3D), consistent with our previous data showing that Mb37_MLKL_ inhibits MLKL function downstream of oligomerization by preventing MLKL membrane association [11]. In addition, like our dox-inducible cell line data (Figures 1 and 2), LNP-delivered Mb37_MLKL_ completely shut down necroptotic cell death in HT29, Colo205 and SW620 cells, while control Mb32_MLKL_ did not (Figure 3E). Importantly, LNP-delivered MLKL intrabodies did not block apoptosis induced by BH3-mimetic, ABT737 and S63845, treatment (Supplementary Figure S4).

### LNP-delivery ASC intrabody mRNA blocks pyroptotic responses

**Figure 4:**
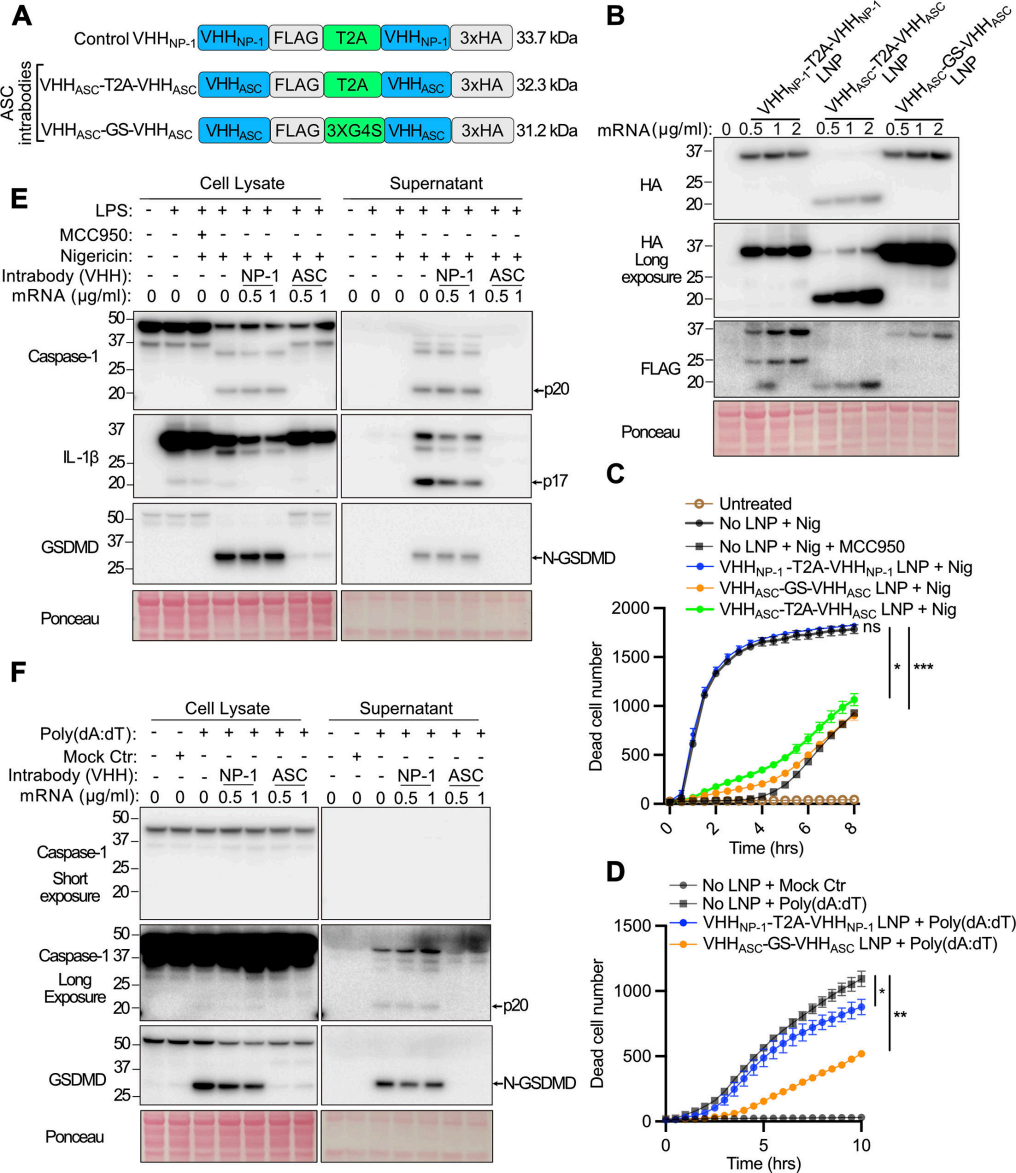
LNP-delivered ASC intrabody mRNA limits pyroptosis. (**A**) A schematic of mRNA constructs. (**B**) Immunoblot analysis of whole-cell lysates harvested after LNP-mediated treatment overnight at the specified mRNA concentrations followed by nigericin (5 µM) stimulation for 1 hour. (**C and D**) iBMDMs were treated overnight with LNPs (0.5 µg/ml mRNA equivalent) and then stimulated with (**C**) nigericin (5 µM) with our without MCC950 (10 µM), or transfected with (**D**) poly(dA:dT) (1 µg/mL). Cell death was measured by IncuCyte imaging. Error bars represent the SD of three technical replicates, *P*≤0.01 (*), *P*≤0.01 (**), *P*≤0.001 (***), two-way ANOVA followed by Tukey’s multiple comparisons test. (**E and F**) Immunoblot analysis of total lysates from iBMDMs pre-treated with increasing concentrations of LNP-mRNA overnight followed by (**E**) LPS (50 ng/ml) priming for 3 hours and then nigericin (5 µM) treatment for 1 hour with or without MCC950 (10 µM), or (**F**) poly(dA:dT) (1 µg/ml) transfection for 6 hours. All data (B to F) are representative of two independent experiments. ASC, apoptosis-associated speck-like protein containing a CARD; LNP, lipid nanoparticle.

Our stable doxycycline-inducible ASC intrabodies showed that VHH_mASC_-T2A-VHH_mASC_ was superior at inhibiting pyroptosis when compared with a single VHH_mASC_ (Figure 2). We therefore generated LNPs containing both self-separating VHH_mASC_-T2A-VHH_mASC_ mRNA and, as a comparison, non-cleavable bivalent VHH_mASC_-GS-VHH_mASC_ mRNA (Figure 4A, Supplementary Figure S2 and **Supplementary Table S1)**. iBMDMs treated with LNP-mRNAs showed robust ASC intrabody protein expression, including excellent separation of VHH_mASC_-T2A-VHH_mASC_ into monomeric intrabodies (Figure 4B). By comparison, however, control VHH_NP-1_-T2A-VHH_NP-1_ separation was poor despite the full-length protein being easily detected (Figure 4B). Regardless, both bivalent and self-separating ASC intrabodies, but not control VHH_NP-1_-T2A-VHH_NP-1_, protected iBMDMs from nigericin-induced pyroptosis comparably, and to a similar extent as MCC950 (Figure 4C). LNP-delivered bivalent ASC intrabody mRNA also protected cells from AIM2-induced pyroptosis (Figure 4D). Consistent with these data, and akin to dox-inducible ASC intrabody expression (Figure 2D and E), LNP-delivered bivalent ASC intrabody mRNA sufficed to inhibit NLRP3- and AIM2-driven caspase-1, IL-1β and GSDMD activation, as reflected by the inhibition of processing into their p20, p17 and pore-forming N-GDSMD fragments, respectively (Figure 4E and F).

## Discussion

Overall, this study presents a clinically relevant small molecule alternative approach for inhibiting intracellular proteins. By deploying intrabody mRNA encapsulated in LNPs as agents to block key proteins required for cellular necrosis, MLKL and ASC, we demonstrate substantial inhibition of inflammatory cell death *in vitro* and show that, in the case of ASC targeting, bivalent ASC intrabody, or self-separating tandem ASC intrabody fusions, exhibit superior protection against pyroptosis when compared with a single ASC nanobody.

Antisense oligonucleotides (ASOs) and siRNAs have been successfully used to silence disease-relevant gene expression, leading to several clinically approved therapies [37,38]. However, protein-targeting intrabodies may offer distinct advantages. Unlike siRNAs and ASOs, which act at the mRNA level, intrabodies target the existing protein pool and will be effective even when the target protein is long-lived. Moreover, intrabodies can be engineered with antibody-like affinity and high functional specificity, enabling recognition of post-translational modifications or conformational states. When fused to E3 ubiquitin ligases, they can also act as ‘biodegraders’ to selectively eliminate target proteins via degradation [7]. Although LNP delivery of both siRNAs and intrabody mRNAs may elicit innate immune responses requiring mitigation, intrabodies such as nanobodies also benefit from millions of years of single-domain antibody evolution, allowing for rapid generation of highly selective binders with minimal off-target effects.

It has previously been shown that purified VHH_mASC_ protein can enter damaged membranes caused by bacterial pore-forming toxins to shut down inflammasome responses [32]. Remarkably, the injection of VHH_mASC_ protein into mice also limited gout-associated monosodium urate (MSU) crystal driven pathology as well as arthritis induced by methylated bovine serum albumin (mBSA) and Freund’s adjuvant [32]. This therapeutic effect may stem from the ability of VHH_mASC_ protein to gain intracellular access via MSU crystal- or mBSA-damaged plasma membrane to block inflammasome activity. Alternatively, or in addition, it may inhibit extracellular polymerized ASC specks that are released following cell lysis and can amplify inflammasome triggering through phagocytic uptake [39,40]. In contrast, because LNP-mRNA-delivered VHH_mASC_ (and Mb37_MLKL_) does not rely on plasma membrane damage to gain access to cytosolic inflammasomes, LNP-mRNA technology may represent a more efficient means to shut down both pyroptosis and inflammasome-driven inflammation, particularly in disease settings that do not involve membrane-damaging microparticles or pore-forming agents. However, efficient LNP-mRNA delivery to relevant target cells and the mitigation of potential LNP-mRNA immunogenicity remains an important area to address for the *in vivo* application of LNP-delivered anti-necrotic intrabody mRNAs.

Our observed functional parity between LNP-delivered intrabody mRNA and those expressed via a doxycycline inducible system underscores the robustness of this delivery modality, further supported by recent studies using LNP-mRNA technology to deliver anti-microbial and anti-cancer nanobodies [41–43]. Altogether, these findings lay the groundwork for advancing LNP-encapsulated intrabody mRNAs into relevant pre-clinical animal disease models, potentially extending antibody therapeutics to intracellular targets and providing a small-molecule alternative for directly drugging disease-causing proteins.

## Materials and methods

### Cell culture

HT29 and HEK293T cells, as well as NLRP3-FLAG/ASC-mCherry-expressing iBMDMs [44], were cultured in DMEM (Gibco, Cat# 11885–084) supplemented with 10% (v/v) foetal bovine serum (FBS; Sigma, Cat# F9423-500ML or Gibco, Cat# 10099–141) and antibiotics (100 U/ml penicillin, and 100 µg/ml streptomycin), at 37°C in a humidified incubator with 10% CO₂. Colo205 and SW620 cells were maintained in RPMI-1640 (Gibco, Cat# 31800089) supplemented with the 10% (v/v) FBS under identical conditions. While the cell lines were not formally authenticated via genetic sequencing, their morphologies and responses to stimuli were consistent with their reported identities.

### Plasmid construction and lentiviral generation of stable cell lines

Constructs were cloned into a dox-inducible pF TRE3G PGK puro plasmid [45] (Supplementary Table S2 and Supplementary Table S3). Constructs were either purchased from Genscript or assembled in-house using the NEBuilder HiFi DNA Assembly Cloning Kit (NEB, Cat# E5520S) and gBlocks Gene Fragments (IDT). Final plasmids were sequence-verified by Sanger sequencing. Full-length plasmid sequences can be provided upon request.

Lentiviral particles were produced by co-transfecting HEK293T cells with packaging and envelope plasmids (pVSVg, pMDL and pRSV-REV) using Lipofectamine 3000 (Thermo Fisher, Cat# L3000008). Viral supernatants were harvested at 48- and 72 hours post-transfection, filtered through a 0.45  µm syringe filter (Sartorius Cat# 6533K) and used to transduce target cells in the presence of 8  µg/ml polybrene (Sigma, Cat# 107689). Successfully transduced cells were selected with 1 µg/ml of puromycin (InvivoGen, Cat# ant-pr-5).

### IVT plasmid and mRNA generation

Gene blocks were amplified via PCR using primers containing NcoI-HF and XhoI (NEB, Cat# R3193 and R0146) restriction sites (Supplementary Table S2). PCR products were gel-purified and ligated into IVT plasmid pre-digested with NcoI and NotI. Ligation reactions were performed using T4 DNA ligase (Promega, Cat# M1801), and the constructs were transformed into DH10α competent *E. coli* by heat shock. Colonies were selected on ampicillin (100 µg/ml)-containing agar plates, and clones positive for insert and polyA were validated by Sanger sequencing and restriction digest analysis with SapI and XbaI (NEB, Cat# R0569 and Cat# R0145), respectively. Midipreps (ZymoPURE II Plasmid Midiprep Kit, Cat# D4200) were performed for the correct clones to isolate plasmid DNA for IVT.

For IVT, plasmids (Supplementary Table S3) were linearized using SapI (NEB, Cat# R0569) and purified using the QIAquick PCR Purification Kit (Qiagen Cat# 28104). Uncapped RNA was synthesized using the HiScribe T7 High Yield RNA Synthesis Kit (NEB, Cat# E2040), while capped RNA was synthesized using the HiScribe T7 mRNA Kit with CleanCap® Reagent AG (NEB, Cat# E2080). A one-pot Cap-1 structure synthesis was performed using Faustovirus capping enzyme and 2′-O-methyltransferase (Company, Cat# M2081, #M0366). After each step, RNA was purified using the Monarch RNA Cleanup Kit (NEB, Cat# T2050). RNA quality was assessed using a Nanodrop, Qubit™ RNA broad range kit (Thermo Fisher, Cat# Q10210), and Tapestation (Agilent Cat# 5067–5576). Capped mRNA was stored at –80°C until use.

### LNP synthesis

mRNA-loaded LNPs were synthesized using a one-step microfluidic mixing protocol on the NanoAssemblr Ignite system. Lipids—SM102 (ionizable lipid), DSPC (zwitterionic lipid), cholesterol and DMG-PEG 2000 – were prepared as 10  mM solutions in ethanol and mixed in a molar ratio of 50:10:38.5:1.5. The mRNA was diluted in 50  mM sodium acetate buffer (pH 4.0) to achieve an aqueous-to-ethanol flow rate ratio of 3:1. The N/P ratio was set at 6, and the mixing was performed at a total flow rate of 12  ml/min. Synthesized LNPs were diluted 30-fold of ethanol volume in DPBS (pH 7.4) and concentrated using 100  kDa centrifugal filters (Millipore, Cat# UFC9100) at 1,500 *g* for 20 minutes. Particle size and PDI were determined by DLS following dilution (5  µL in 45  µL PBS). RNA encapsulation efficiency was measured using the Qubit RNA Broad-Range Assay after treating LNPs with either TE buffer or TE containing 2% Triton X-100. The encapsulated RNA content was calculated by subtracting the untreated (TE) signal from the lysed (Triton-treated) signal, expressed as a percentage of the input RNA.

### Immunoblotting

Cells were seeded into 6- or 12-well tissue culture-treated plates (Corning®, Cat #3506 and #3512) and incubated for 24 hours before dox induction (100  ng/mL) or treatment with LNPs containing 1 µg/ml or 0.5 µg/ml of mRNA equivalent, alone or in combination with other drugs, as specified in relevant figure legends. Cells were lysed in 2 × SDS Laemmli buffer, heated at 95°C for 10–15 minutes, and proteins separated using 4%–12% gradient Bis-Tris SDS-PAGE gels (Invitrogen, Cat# NP0321). Proteins were transferred onto nitrocellulose membranes (Amersham, Cat# GE10600073). Ponceau S staining was routinely performed to verify equal protein loading. Membranes were blocked with 5% (w/v) skim milk in TBS containing 0.1% Tween 20 (TBS-T) and probed overnight at 4°C with primary antibodies in blocking buffer with 0.04% sodium azide. Horseradish peroxidase-conjugated secondary antibodies were incubated for 1 hour at room temperature. Blots were developed using ECL (Millipore, Bio-Rad) and imaged using the ChemiDoc Touch Imaging System (Bio-Rad). Antibody details are provided in the Supplementary Table S4.

### IncuCyte cell death assays

Colo205, SW620 and HT29 cells were plated at 3,000 cells/well in 96-well plates and allowed to adhere for 24 hours. Cells were pre-treated with doxycycline (100  ng/ml) overnight prior to the addition of reagents, including TNF (50  ng/ml), Smac mimetic Compound A (1  µM), pan-caspase inhibitor IDN-6556 (10  µM), ABT737 (1  µM), and S63845 (10  µM), alone or in combination. For iBMDMs, cells were plated at 10,000 cells/well in 96-well plates and allowed to adhere for 24 hours. Cells were pre-treated with doxycycline (100  ng/ml) overnight prior to the addition of reagents including nigericin (5  µM, Sigma, Cat# N7143), or Val-boroPro (VbP, 2.5 µM, InvivoGen, Cat# tlrl-vbp), or transfection of poly(dA:dT) (InvivoGen, Cat# tlrl-patn) using Lipofectamine 3000. Cell death was assessed using propidium iodide (PI, 0.2  µg/mL), SYTOX Green (0.5  µM, Thermo Fisher and cat# S7020) or TO-PRO-3 Stain (Thermo Fisher and cat#T3605, NIR dead cell dye, 1:5000 dilution) combined with nuclear dyes SPY505, SPY595, or SPY700 (Spirochrome, Cat# SPY505-DNA, SPY595-DNA, or SPY700-DNA). Images were captured using the IncuCyte SX5 or S3 imaging system (software versions v2022B or v2021B). The percentage of cell death was calculated by dividing the number of TO-PRO-3, PI or SYTOX Green-positive cells (dead) by the total number of SPY-positive cells (live and dead).

## Supplementary Material

Online supplementary material 1

## Data Availability

All supporting data are included in the main article and its supplementary file. Reagents and materials are available upon request via contacting the corresponding author, James E Vince.

## Abbreviations

ASC: apoptosis-associated speck-like protein containing a CARD
IVT: *in vitro* transcription
LNP: lipid nanoparticle
MLKL: mixed lineage kinase domain-like pseudokinase
