# Supplementary material for: Lipid nanoparticle-delivered intrabodies for inhibiting necroptosis and pyroptosis
Source: Biochem J. 2025 Aug 20;482(16):1197–208. doi: 10.1042/BCJ20253191 (PMC12493153; doi:10.1042/BCJ20253191)
Supplement: Online supplementary material 1 [file bcj-482-16-BCJ20253191-s001.pdf]

Supplemental Figures and Tables

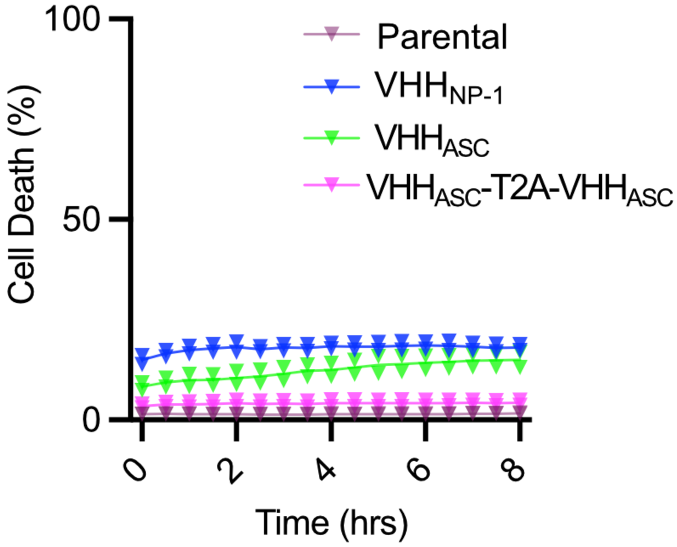

Figure S1. Intrabody expression alone does not cause cell death. Wildtype parental and the indicated stable iBMDM lines were treated overnight with 0.1 µg/mL doxycycline and cell death analysed. Represents control data (*i.e.,* no nigericin treatment) for Figure 2A.

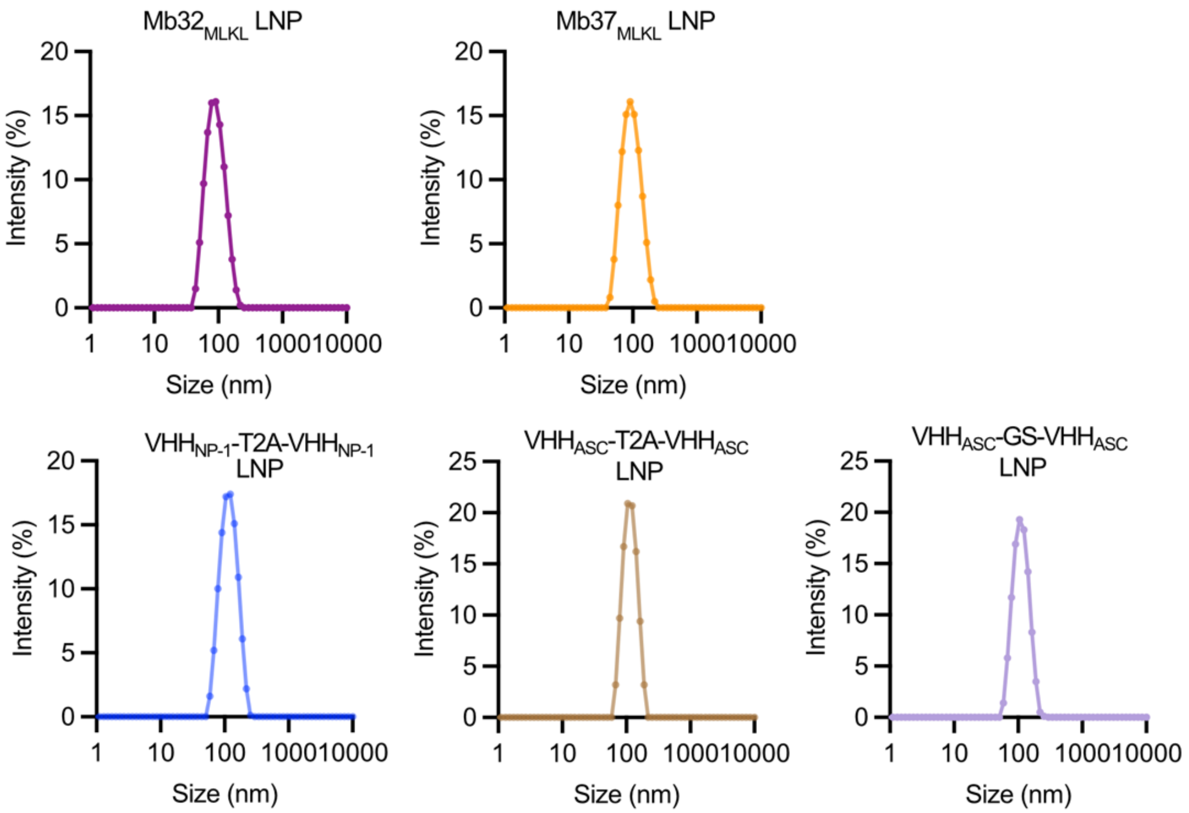

Figure S2. Unimodal LNP-mRNA size. Size distribution of LNP-mRNAs as measured by dynamic light scattering.

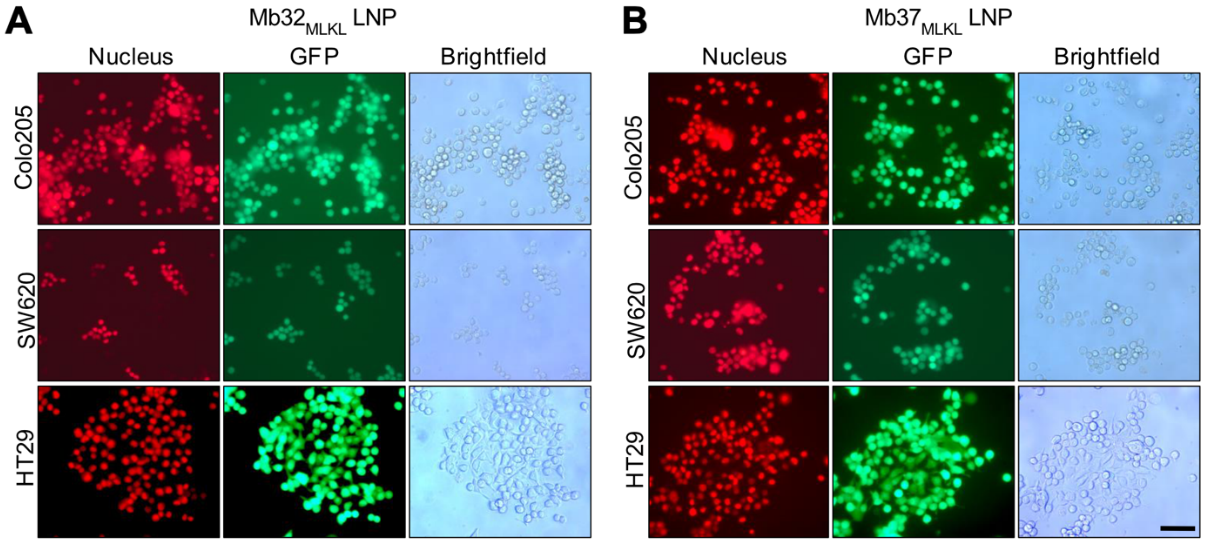

Figure S3. LNP-delivered MLKL intrabodies are expressed in the majority of cells. Widefield fluorescence microscopy recorded 24 hours after LNP-mRNA treatment 0.5 µg/mL mRNA equivalent in the indicated cancer cell lines. Nuclei were stained with SPY DNA 620. Scale bar, 200 µm.

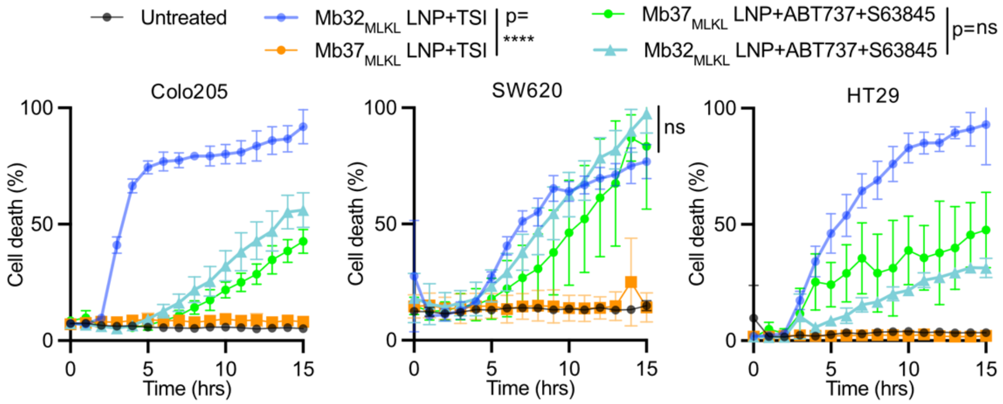

Figure S4. MLKL intrabodies do not block intrinsic apoptosis. Cell death kinetics measured by IncuCyte live-cell imaging following treatments to induce either necroptosis or apoptosis. Necroptosis was triggered using a TSI stimuli consisting of TNF (50 ng/mL), Smac mimetic Compound A (1 µM), and the pan-caspase inhibitor IDN-6556 (10 µM). Intrensic apoptosis was induced using ABT-737 (1 µM) and S63812 (5 µM). Prior to cell death stimuli, cells were pretreated overnight with $Mb32_{MLKL}$ LNP or $MB37_{MLKL}$ LNPs at 0.5 µg/mL mRNA equivalent. Error bars represent the standard deviation of three technical replicates, $p \leq 0.0001$ (****), significance calculated via a one-way ANOVA followed by Tukey's multiple comparisons test. The data are representative of two independent experiments.

Table S1. Physiochemical characterisation of LNP-mRNAs.

LNP	Z-average diameter (nm)	PDI	mRNA loading efficiency (%)
$Mb32_{MLKL}$ LNP	88.7	0.13	87.4
$Mb37_{MLKL}$ LNP	85.6	0.11	89.2
$VHH_{NP\text{-}1}$-T2A-$VHH_{NP\text{-}1}$ LNP	106.0	0.054	85.6
VHH_{mASC}-GS-VHH_{mASC} LNP	110.3	0.045	86.4
VHH_{mASC}-T2A-VHH_{mASC} LNP	110.7	0.066	89.4

Table S2. Sequence of primers and gene blocks.

Primer/gene block	Sequence
**Primer**	
Forward primer for FLAG-Mb32$_{MLKL}$-GFP and FLAG-Mb37$_{MLKL}$ -GFP	5'-<br>TGACCCATGGTCTAGACCACCATGGATTACAAAGACGATG<br>ATGAT-3'
Reverse primer for FLAG-Mb32$_{MLKL}$ -GFP and FLAG-Mb37$_{MLKL}$ -GFP	5'-TTATCTCGAGTTACTTGTACAGCTCGTCCATGCC-3'
**Gene block**	
FLAG-Mb32 $_{MLKL}$-GFP	5'-CACCATGGATTACAAAGACGATGATGATAAGGGATCCGT<br>TTCTTCTGTTCCGACCAAACTGGAAGTTGTTGCTGCGACCC<br>CGACTAGCCTGCTGATCAGCTGGGATGCTCCTGCAGTTACC<br>GTCGATCTTTACATTATCACGTACGGTGAAACCGGTGGTAAC<br>TCCCCGGTTCAAACGTTTGAGGTACCAGGTTCCAAGTCTAC<br>TGCTACCATCAGCGGCCTGAGCCCGGGTGTCGACTATACCA<br>TCACTGTATACGCATACTCTTTCATGTACCATGACTACTACTA<br>CCCGGAATGGAGCCCAATCTCGATTAACTACCGTACCGCTA<br>GCAGTTCCTCTAGTGCGGCCGCTGTGAGCAAGGGCGAGGA<br>GCTGTTCACCGGGGTGGTGCCCATCCTGGTCGAGCTGGACG<br>GCGACGTAAACGGCCACAAGTTCAGCGTGTCCGGCGAGGG<br>CGAGGGCGATGCCACCTACGGCAAGCTGACCCTGAAGTTC<br>ATCTGCACCACCGGCAAGCTGCCCGTGCCCTGGCCCACCCT<br>CGTGACCACCCTGACCTACGGCGTGCAGTGCTTCAGCCGCT<br>ACCCCGACCACATGAAGCAGCACGACTTCTTCAAGTCCGC<br>CATGCCCGAAGGCTACGTCCAGGAGCGCACCATCTTCTTCA<br>AGGACGACGGCAACTACAAGACCCGCGCCGAGGTGAAGTT<br>CGAGGGCGACACCCTGGTGAACCGCATCGAGCTGAAGGGC<br>ATCGACTTCAAGGAGGACGGCAACATCCTGGGGCACAAGC<br>TGGAGTACAACTACAACAGCCACAACGTCTATATCATGGCC<br>GACAAGCAGAAGAACGGCATCAAGGTGAACTTCAAGATCC<br>GCCACAACATCGAGGACGGCAGCGTGCAGCTCGCCGACCA<br>CTACCAGCAGAACACCCCCATCGGCGACGGCCCCGTGCTG<br>CTGCCCGACAACCACTACCTGAGCACCCAGTCCGCCCTGA<br>GCAAAGACCCCAACGAGAAGCGCGATCACATGGTCCTGCT<br>GGAGTTCGTGACCGCCGCCGGGATCACTCTCGGCATGGAC<br>GAGCTGTACAAGTAACTC-3'
FLAG-Mb37$_{MLKL}$-GFP	5'-CCATGGATTACAAAGACGATGATGATAAGGGATCCGTTTC<br>TTCTGTTCCGACCAAACTGGAAGTTGTTGCTGCGACCCCGA

	CTAGCCTGCTGATCAGCTGGGATGCTCCTGCAGTTACCGTC<br>GATTATTACTATATCACGTACGGTGAAACCGGTGCTTCTTTC<br>TACTTCTACCAGACATTCAAGGTACCTGGTTCCAAGTCTACT<br>GCTACCATCAGCGGCCTGAGCCCGGGTGTCGACTATACCAT<br>CACTGTATACGCATACGACTGGGGTGGTTACTTCATCGGTAA<br>CCCAATCTCGATTAACTACCGTACCGCTAGCAGTTCCTCTAG<br>TGCGGCCGCTGTGAGCAAGGGCGAGGAGCTGTTCACCGGG<br>GTGGTGCCCATCCTGGTCGAGCTGGACGGCGACGTAAACG<br>GCCACAAGTTCAGCGTGTCCGGCGAGGGCGAGGGCGATGC<br>CACCTACGGCAAGCTGACCCTGAAGTTCATCTGCACCACCG<br>GCAAGCTGCCCGTGCCCTGGCCCACCCTCGTGACCACCCTG<br>ACCTACGGCGTGCAGTGCTTCAGCCGCTACCCCGACCACAT<br>GAAGCAGCACGACTTCTTCAAGTCCGCCATGCCCGAAGGC<br>TACGTCCAGGAGCGCACCATCTTCTTCAAGGACGACGGCA<br>ACTACAAGACCCGCGCCGAGGTGAAGTTCGAGGGCGACAC<br>CCTGGTGAACCGCATCGAGCTGAAGGGCATCGACTTCAAG<br>GAGGACGGCAACATCCTGGGGCACAAGCTGGAGTACAACT<br>ACAACAGCCACAACGTCTATATCATGGCCGACAAGCAGAA<br>GAACGGCATCAAGGTGAACTTCAAGATCCGCCACAACATC<br>GAGGACGGCAGCGTGCAGCTCGCCGACCACTACCAGCAGA<br>ACACCCCCATCGGCGACGGCCCCGTGCTGCTGCCCGACAA<br>CCACTACCTGAGCACCCAGTCCGCCCTGAGCAAAGACCCC<br>AACGAGAAGCGCGATCACATGGTCCTGCTGGAGTTCGTGA<br>CCGCCGCCGGGATCACTCTCGGCATGGACGAGCTGTACAAG<br>TAACTC-3’
VHH$_{mASC}$-T2A-VHH$_{mASC}$	5’-ATAACGCGTCCATTCGACACGCCACCATGGCTCAGGTGC<br>AGCTGGTGGAGACTGGGGGCGGAATGGTCCATCCCGGTGG<br>CAGCCTGCGCCTGAGCTGTGCAGCCAGTGGCTTTACTTTCA<br>GCGAGTACGGGATGACTTGGGTGAGACAGGCTCCCGGGAA<br>GGGCCCCGAATGGGTGTCCAGGATTAACAGCAGTGGCGGA<br>TATACCGTATACAGGGCCTCTGTGAAAGGACGGTTTACCGT<br>GAGCAGAGACAACGCCAAGAACACCCTATACCTTCAGATG<br>AATTCACTGAAGCCAGAGGACACAGCCCTGTATTACTGTGC<br>CCGGACCACTAACTGGGAGACCAGACTATCTCAGGGCACC<br>CAGGTTACTGTCAGCAGCGGAGGTGACTACAAGGATGATG<br>ACGACAAGGGAGGGGGCGGCTCAGGAGGGGGGGTAGTG<br>GTGGTGGTGGCTCTCAGGTCCAGCTTGTGGAAACAGGAGG<br>AGGCATGGTGCACCCTGGTGGGTCACTGCGTCTGTCCTGCG<br>CCGCCTCCGGGTTCACCTTCTCTGAGTATGGCATGACCTGG<br>GTAAGACAAGCACCTGGCAAAGGCCCTGAGTGGGTTTCCA<br>GAATCAATTCCTCCGGCGGTTACACAGTCTACAGAGCTAGT<br>GTGAAGGGACGCTTCACGGTGAGCAGAGACAATGCCAAAA<br>ACACATTGTACCTGCAGATGAACTCATTAAAGCCAGAAGAC<br>ACAGCTTTGTACTACTGCGCCAGGACCACAAACTGGGAAA<br>CCAGACTGAGCCAAGGCACACAGGTGACAGTAAGCAGTGG<br>CGGTTACCCTTATGATGTGCCTGACTATGCTGGCTACCCATAT

	GACGTGCCAGACTACGCAGGTTCCTACCCCTACGATGTTCC<br>TGATTATGCCTGAGCGGCCGCTCGAGGCTCGCTTTCTTGCT<br>GTC-3’
VHH_{NP1}-T2A-VHH_{NP1}	5’ATAACGCGTCCATTCGACACGCCACCATGGCTCAAGTACA<br>GCTCCAGGAAAGCGGTGGTGGACTCGTTCAAGCCGGGGGG<br>TCTCTGCGCCTTACTTGCGCACTTTCCGAGCGAACCAGCAC<br>TTCATACGCCCAGGGGTGGTTCAGACAGCCTCCCGGCAAG<br>GAGAGAGAATTCGTCGCAAGCCTCCGAACACATGATGGTA<br>ACACACACTATACCGATTCAGTGAAAGGTCGGTTTACAATAT<br>CTAGGGATAATGCCGAAAATACATTGTATCTCCAGATGAACT<br>CTCTTAAAACAGAAGATACAGCTGTGTACTACTGTGCCGCT<br>TCTCTCGGTTACTCAGGTGCTTACGCAAGCGGGTATGACTA<br>CTGGGGCCAAGGGACACAAGTTACAGTAAGCAGCGGTGGA<br>GATTACAAAGACGACGATGACAAGGGATCAGGCGAAGGCC<br>GGGGCAGCCTGCTCACCTGCGGAGATGTAGAAGAGAACCC<br>TGGGCCCCAGGTACAGCTGCAGGAATCAGGAGGGGGCCTG<br>GTGCAGGCTGGAGGGTCCCTTAGGCTGACATGCGCCCTCTC<br>TGAGAGGACCAGTACCTCATACGCTCAGGGCTGGTTTAGAC<br>AGCCACCCGGGAAGGAACGCGAGTTTGTCGCTTCCCTGCG<br>CACCCACGACGGAAATACTCATTATACAGATAGTGTTAAGG<br>GTCGGTTCACCATCAGTAGGGATAACGCTGAAAATACATTG<br>TACCTTCAAATGAATTCACTCAAGACCGAAGATACAGCAGT<br>CTACTATTGCGCAGCTAGTTTGGGTTACAGCGGAGCTTATGC<br>AAGTGGTTATGATTACTGGGGGCAGGGAACTCAGGTTACTG<br>TATCCAGTGGGGGTTATCCATATGATGTTCCAGACTATGCAG<br>GATATCCATACGATGTGCCCGACTACGCCGGCTCCTATCCAT<br>ACGACGTGCCAGATTATGCCTGAGCGGCCGCTCGAGGCTCG<br>CTTTCTTGCTGTC-3’

Table S3. Plasmids used to make stable cell lines and IVT templates.

**Plasmid**	**Description**	**reference**
Doxycycline inducible plasmids		
pF TRE3G_FLAG-MB32$_{MLKL}$-GFP<br>pF TRE3G_FLAG-MB37$_{MLKL}$-GFP	MLKL inhibitory monobody (Mb37) or control non-inhibitory monobody (Mb32)	Petrie, E. J. *et al*, 2020[1].
pF_TRE3G_VHH$_{NP-1}$-3xHA	Control NP-1 nanobody	This work, Genscript
pF TRE3G_VHH$_{mASC}$-3xHA	ASC nanobody	This work, Genscript
pF TRE3G VHH FLAG-VHH$_{mASC}$-T2A_VHHm$_{ASC}$-3xHA	Self-separating ASC nanobody	This work, Genscript
IVT plasmids		
pGEMs_eGFP_IVaxT_Poly_A_148	Parental IVT plasmid	Fernández, S.L. *et*, 2021[2].
pGEMs_VHH$_{NP-1}$-FLAG-T2A-VHH $_{NP-1}$-3xHA_IVT CleanCap	Control self-separating NP-1 nanobody	This work, made in house
pGEMs_VHH$_{mASC}$-FLAG-T2A-VHH$_{mASC}$-3xHA_IVT CleanCap	Self-separating ASC nanobody	This work, made in house
pGEMs_VHH$_{mASC}$-FLAG-3xG4S-VHH$_{mASC}$-3xHA_IVT CleanCap	Bivalent ASC nanobody	This work, made in house
pGEMs_ FLAG-MB32$_{MLKL}$-GFP_IVT	Control MLKL monobody	This work, made in house
pGEMs_ FLAG-MB37$_{MLKL}$-GFP_IVT	Inhibitory MLKL monobody	This work, made in house

1 Petrie, E. J., Birkinshaw, R. W., Koide, A., Denbaum, E., Hildebrand, J. M., Garnish, S. E., Davies, K. A., Sandow, J. J., Samson, A. L., Gavin, X., Fitzgibbon, C., Young, S. N., Hennessy, P. J., Smith, P. P. C., Webb, A. I., Czabotar, P. E., Koide, S. and Murphy, J. M. (2020) Identification of MLKL membrane translocation as a checkpoint in necroptotic cell death using Monobodies. Proceedings of the National Academy of Sciences. **117**, 8468-8475

2 Linares-Fernandez, S., Moreno, J., Lambert, E., Mercier-Gouy, P., Vachez, L., Verrier, B. and Exposito, J. Y. (2021) Combining an optimized mRNA template with a double purification process allows strong expression of in vitro transcribed mRNA. Mol Ther Nucleic Acids. **26**, 945-956

Table S4. Antibodies.

Antibody	Company/catalogue number	Dilution
Anti-FLAG M2-Peroxidase (HRP) antibody	Sigma-Aldrich/Cat# A8592	1:4000
Rat anti-MLKL	Inhouse;<br>available from Merck Millipore / Cat# MABC604	1:1000
Rabbit anti-MLKL (phospho S358)	Abcam/ Cat# ab187091	1:1000
Rabbit anti-MCL-1	Cell Signaling Technology/Cat# 5453	1:1000
Rabbit anti-GFP	Invitrogen/Cat# A-6455	1:2000
Mouse anti-VDAC1	Merck Millipore/MABN504	1:500
Rabbit anti-GAPDH	Cell Signaling Technology/Cat# 2118	1:1000
Rabbit anti-ASC	Adipogen/Cat# AL177	1:1000
Mouse anti-NLRP3	Adipogen/ AG-20B-0014-C100	1:1000
Mouse anti-IL1β	R&D/ Cat# AF-401-NA	1:1000
Mouse anti-Caspase 1	Adipogen/ Cat# AG-20B-0042-C100	1:1000
Rabbit anti-Caspase 1	Abcam/ Cat# ab179515	1:1000
Rabbit anti-GSDMD	Abcam/ Cat# ab209845	1:1000
HRP-conjugated anti-rabbit secondary	Southern Biotech/ Cat# 4010-05	1:10000
HRP-conjugated anti-mouse secondary	Southern Biotech/ Cat# 1010-05	1:10000
HRP-conjugated anti-rat secondary	Southern Biotech/ Cat# 3010-05	1:10000
